# Experimental Study on the Dynamic Fracture Characteristics of Mortar–Rock Interface Zones with Different Interface Inclinations and Shapes

**DOI:** 10.3390/ma16155475

**Published:** 2023-08-04

**Authors:** Zhaoqi Li, Jie Dong, Tao Jiang, Kai Feng, Siwu Cheng, Yuqian Liu, Guoxiang Zhang, Xuewei Tian

**Affiliations:** 1 College of Civil Engineering, Hebei University of Architecture, Zhangjiakou 075000, China; li592890051@outlook.com (Z.L.); cswcsw1651049@outlook.com (S.C.); liutian970219@outlook.com (Y.L.); 2 Hebei Colleges Applied Technology Research Center of Green Building Materials and Building, Reconstruction, Zhangjiakou 075000, China; 3 China Railway Design Group Limited, Tianjin 300380, China

**Keywords:** mortar–rock interface, dynamic fracture characteristics, inclination angle, failure modes, dissipated energy

## Abstract

There has been little research on the impact resistance of mortar–rock slope protection structures. To ensure that the mortar–rock interface has good adhesion properties under the action of impact loading, in this paper, based on fracture mechanics theory, a theoretical impact model was established for mortar–rock binary material. Dynamic fracture tests were carried out on mortar–rock interfaces using the split-Hopkinson pressure bar (SHPB) system. The Brazilian disc (CSTBD) specimen was prepared with one half in granite and the other half in mortar. The specimen used for the dynamic impact test was 48 mm in diameter and 25 mm thick. The effects caused by the change in interface inclination and interface shape on the dynamic fracture mode were discussed. The dynamic model parameters were obtained for different inclination angles and interfaces. The results show that both the interface inclination and interface shape have significant effects on the dynamic mechanical properties of the mortar–rock binary material. The fracture modes of the mortar–rock specimens can be classified into three types. When the interface inclination is 0°, the specimen shows shear damage with an interface fracture; when the interface inclination is in the range of 0–90°, the dynamic splitting strength of the mortar–rock material increases with increasing interface inclination, and the interface undergoes composite fracture; and when the interface inclination is 90°, the dynamic splitting strength of the specimen reaches its peak, and the interface undergoes tensile fracture. The mortar–rock interface damage follows the M-C criterion. The roughness of the interface shape has a large influence on the dynamic splitting strength of the specimens. The rougher the interface shape, the higher the interface cleavage strength and the higher the peak load that causes the material to damage. The results of this study can provide a reference for the design of mortar–rubble structures to meet the demand for impact resistance and have strong engineering application value.

## 1. Introduction

With the development of bridge construction in China, the number of large-span bridges built in mountainous areas is increasing. Mortar–rubble retaining slopes are often used in tapered slope support projects at the ends of bridges in mountainous areas [1]. As a mortar–aggregate masonry retaining wall, the mortar–rubble protection structure is vulnerable to the effects of impact caused by falling rocks, rock bursts or blasting. This seriously affects the normal operation of the retaining structure and causes serious damage to the stability and durability of the structure. It further threatens the operational safety of the connecting traffic arteries. To avoid cracking and destabilisation damage to the conical slope of the bridge abutment, the mortar–rubble structure needs to have adequate impact resistance. Currently, the study of damage to concrete materials under impact loading is an issue of concern [2,3,4,5,6,7,8,9,10,11,12,13,14].

Recently, some scholars have investigated the interface properties in concrete materials from experimental and analytical perspectives [15,16,17,18,19,20,21]. Kaplan [22] was the first to introduce the concept of fracture mechanics in the study of crack extension. A large number of scholars have studied the mode of crack extension in concrete and carried out a series of interfacial splitting tests [23,24,25,26]. However, most of the aforementioned studies were quasistatic experiments conducted on concrete–rock composites at low strain rates. The dynamic fracture behaviour of mortar–rock materials under medium strain rates has not been considered. To ensure good adhesion properties at the mortar–rock interface under impact loading, a study of the dynamic fracture mode of the mortar–rock interface is needed. Among these properties, the dynamic fracture toughness and dynamic tensile strength of the mortar–rock interface are the key to the study of dynamic fracture properties. It is worthwhile to study them in depth.

The dynamic fracture performance of the interface of a bimaterial is the key to its impact resistance. In summary, numerous scholars have carried out a large number of studies on the dynamic fracture mechanism at the interface [27,28,29]. A number of parameters play a crucial role in understanding the dynamic fracture mechanism of binary specimens [30,31,32]. The bonding strength between mortar and rock is a critical parameter affecting the fracture behaviour. A strong bond between the two materials facilitates energy transfer and promotes crack propagation along the interface, resulting in mixed-mode fracture. Zhou et al. [33] carried out disc fracture tests to investigate the fracture mechanisms of bimaterial disc specimens with different inclination angles under two strain rates. Guo et al. [34] discussed the effects of material strength and load rate on the dynamic fracture toughness of a material under different loading modes. Zhong [35] studied specimens with filled crack defects under uniaxial compression and compared them with unfilled specimens. The results show that the crack initiation time, location, and peak strength were highly dependent on the mechanical properties of the filled materials. Wang et al. [36] studied the failure patterns and shear strength of brittle materials containing double-filled flaws under biaxial and triaxial compression. Several authors have used a combination of experimental and numerical calculations to determine the tensile strength of mortar–rock bimaterial specimens [37,38,39,40]. Several scholars have used different nondestructive testing tools to probe the fracture resistance of materials, such as DIC, acoustic emission, and laser scanning. Mpalaskas A.C. [41] performed fracture experiments on granite specimens to compare the typical acoustic emission (AE) signals in different modes (i.e., bending and shear) for smooth granite and marble specimens, as well as for cracked surfaces repaired with polyester adhesive. Kewei Liu [42] used digital image correlation (DIC) to monitor the fracture evolution of rock–concrete bimaterials. The significance of the effect of increasing the interface tilt angle on the static and dynamic fracture toughness of CSTBD specimens was compared. Zhu et al further investigated the effects of rock type, concrete properties (strength and admixtures), interface roughness, and loading rate on the dynamic tensile behaviour of composite rock and concrete specimens by using DIC, laser scanning, and scanning electron microscopy (SEM) techniques [43,44,45,46]. Qiu et al. [47] derived a complex SIF for the material interface using the ABAQUS code, and the corresponding fracture toughness was obtained by comparison with the experimental results. However, the dynamic fracture mechanism of mortar–rock has not been investigated in conjunction with the interface inclination angle and interface shape.

To investigate the influence of aggregate shape and interface inclination on the dynamic mechanical properties of mortar–rock bimaterial specimens, good interfacial bonding properties of mortar–rock bimaterials under impact loading were ensured. In this paper, a theoretical model of the dynamic fracture of mortar–rock bimaterials was developed based on the theory of interfacial fracture mechanics. Dynamic fracture tests on the mortar–rock interface were carried out using a split Hopkins pressure bar (SHPB) device. The output waveform transformation was also optimised using a shaper. This analysis focuses on the effects caused by the change in mortar–rock interface inclination and interface shape on the dynamic fracture mode. The results can be used as a reference for the study of the impact resistance of mortar–rock slope protection structures, since they can help to optimise the design of the mortar–rock slope protection structure in terms of its arrangement and to improve the construction efficiency of mortar–rock slope protection projects.

## 2. Dynamic Fracture Theory of Interfaces

**Figure 1:**
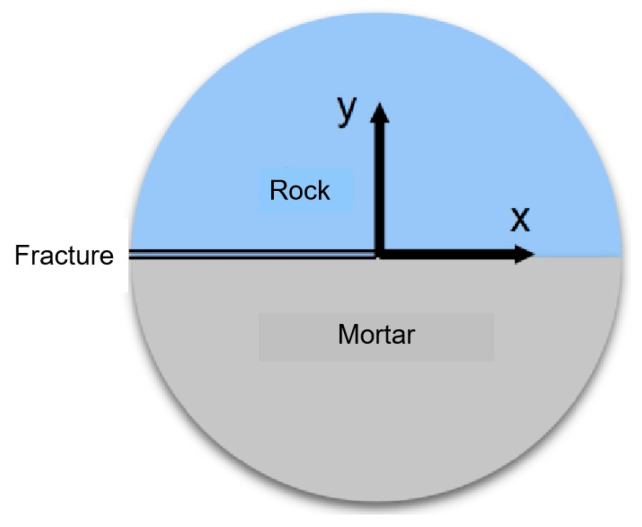
Dynamic fracture mechanics model for a mortar–rock interface.

Fracture toughness plays a key role in the study of the dynamic fracture resistance of the mortar–rock interface and can control the stress intensity factor. According to the dynamic fracture mechanics of an interface, the origin of the polar coordinates is set at the interface joint of the mortar–rock bimaterial, as shown in Figure 1, and the crack tip equation of the mortar–rock bimaterial is solved by elastic mechanics, as shown in Equation (1).

(1) σ=12πr{Re(Kriε)σ1(θ)+Im(Kriε)σ2(θ)}

where $i=\sqrt{-1}$, *r* and *θ* are the polar coordinates, and *σ*_1_ and *σ*_2_ are angular distribution functions.

In dynamic loading, Equations (2) and (3) can be used for the predictive assessment of dynamic stress intensity factors [18,19,20,21,22,23,24,25,26,27,28,29,30,31,32,33,34,35,36,37,38,39,40,41,42,43,44,45,46,47,48,49,50].

(2) $$K_{1}\left(t\right)=\frac{P}{\pi BR}\sqrt{\pi a}Y_{1}$$

(3) $$K_{2}\left(t\right)=\frac{P}{\pi BR}\sqrt{\pi a}Y_{2}$$

where *K*_1_ and *K*_2_ are the real and imaginary parts of the stress intensity factor.

The stress relationship for the tip crack strength factor of the mortar–rock bimaterial is defined as shown in Equation (4).

(4) $${\left(\sigma_{y}+i\sigma_{\mathrm{xy}}\right)}_{\theta =0}=\frac{\left(K_{1}+iK_{2}\right)r^{i\varepsilon }}{\sqrt{2\pi r}}$$

where *r^iε^* is expressed as the oscillatory singular function of the crack. When lim*r* → 0, the tensile and compressive stresses alternate cyclically.

This factor is expressed as

(5) $$\varepsilon =\frac{1}{2\pi }\mathrm{In}\frac{1-\beta }{1+\beta }$$

The energy release *H* can be expressed as

(6) $$H=\frac{1-\beta^{2}}{E^{*}}\left(K_{1}^{2}+K_{2}^{2}\right)$$

(7) $$\frac{1}{G^{*}}=\frac{1}{2}\left(\frac{1}{G_{A}}+\frac{1}{G_{B}}\right)\frac{1}{E^{*}}=\frac{1}{2}\left(\frac{1}{E_{A}}+\frac{1}{E_{B}}\right)\beta =\frac{1}{2}\frac{G_{A}\left(1-2\nu_{B}\right)-G_{B}\left(1-2\nu_{A}\right)}{G_{A}\left(1-\nu_{B}\right)-G_{B}\left(1-\nu_{A}\right)}$$

where *ν_A_* and *ν_B_* are the Poisson’s ratios of the mortar and rock, *G_A_* and *G_B_* are the shear moduli of the mortar and rock, and *E_A_* and *E_B_* are the elastic moduli of the mortar and rock.

In fact, *K*_1_ and *K*_2_ are not stress intensity factors in the two fracture states only but are also closely related to the positive and shear stresses at the crack front. Both describe the law of stress and strain fields affecting the crack tip of a mortar–rock binary structure under impact loading. The crack initiation and development process can be analysed according to this stress intensity factor [49]. The polar coordinates, centred on the crack tip, are defined as

(8) $$\begin{array}{l} K_{1}=\sigma_{xy}\sqrt{2\pi r}\sin\left(\varepsilon \mathrm{In}r\right)+\sigma_{y}\sqrt{2\pi r}\cos\left(\varepsilon \mathrm{In}r\right)\\ K_{2}=\sigma_{xy}\sqrt{2\pi r}\cos\left(\varepsilon \mathrm{In}r\right)-\sigma_{y}\sqrt{2\pi r}\sin\left(\varepsilon \mathrm{In}r\right) \end{array}$$

According to previous studies, the loading fracture mode of cementitious materials tends to be I/II composite cracking. Therefore, the composite stress intensity factor at the interface can be calculated based on the real and imaginary parts of *K*, as in Equation (9).

(9) $$K=\sqrt{K_{1}{}^{2}+K_{2}{}^{2}}$$

## 3. Materials and Methods

### 3.1. Material Characterisation and Specimen Preparation

**Figure 2:**
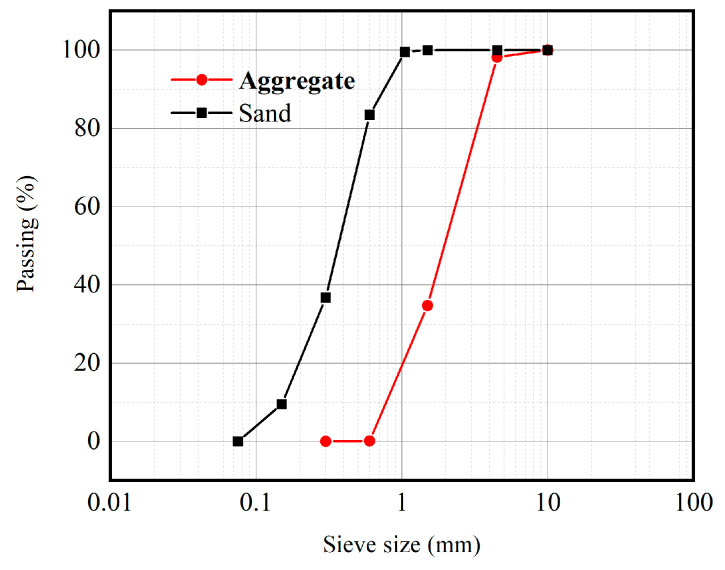
Size gradation curves of aggregates.

Granite aggregates from Jining, Shandong Province, were chosen for the rock portion of this test, and the mechanical parameters of the granite are shown in Table 1. Granite was chosen as the aggregate, mainly because granite has tightly connected internal particles and is commonly encountered in mortar slab slope construction for cold regions. Therefore, granite was chosen to make bimaterial specimens, and the data are more representative. The uniaxial compressive strength of the granite was tested by means of a hydraulic servo press, and the average value was calculated for every group of three specimens. The elastic modulus was calculated from the test results. The type of mortar selected was M10 standard mortar (the compressive strength is 10 Mpa). The admixture was determined to be 2% of the cementitious material, and the water/cement ratio was 0.36. The mortar components are provided in Table 2. The cement was made of P.O 42.5 silicate cement produced by the GTC company. The physical and mechanical parameters of the cement are shown in Table 3. The density of silica fume is 2.3 g/m^3^, and the bulk density is 240 g/m^3^. For sand, natural river sand was used. The physical and mechanical parameters of the sand are given in Table 4. It has a bulk density of 1500 kg/m^3^, water absorption of 1.2%, and a fineness modulus of 2.6. The granite was sampled as an aggregate for X-ray diffraction (XRD) analysis, and the mineral fractions of the granite were analysed to include quartz, sodium feldspar, microcline feldspar, and black mica. The granite components are provided in Table 4. The size gradation curves of aggregates are shown in Figure 2.

In this thesis, tests were carried out using a detached Hopkinson rod of φ48. Following Davis [19], the length/diameter ratio of the mortar–rock specimen for the SHPB (Split Hopkinson Pressure Bar) impact test was calculated according to Equation (10).

(10) $$\frac{L}{D}=\sqrt{\frac{3}{4}v}$$

where *ν* is Poisson’s ratio. The size of the mortar–rock binary specimen for the dynamic impact test was Φ 48 × 25 mm^2^. The diameter of the specimen is 48 mm, and the thickness is 24 mm. The binary specimen of granite and mortar was prepared by the following procedure. The aggregate was processed using a cutting machine to cut out the complete interface shape. The interface was cut to improve the roughness of the interface and to enhance the bond between the mortar and the rock interface. An acrylic tube was cut into acrylic rings of Φ 48 mm × 25 mm^2^ and sealed with an equal diameter round cap of 2 mm thickness with waterproof tape, repeatedly shaped to ensure the flatness of the bottom. Machine oil was applied to the inner wall and bottom of the acrylic mould to facilitate the release of the specimen from the mould. The mortar was poured into the acrylic moulds in proportion to the mix design. The moulds were then released after 1 day. The specimens were cured for 28 d under standard curing conditions. The specimens were taken out of the curing room, and the top and bottom surfaces were polished to within ±0.02 mm.

### 3.2. Experimental Apparatus

**Figure 3:**
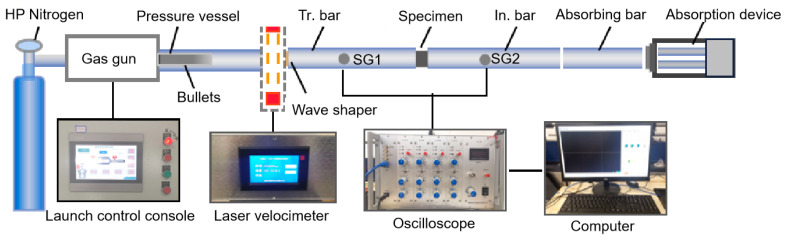
The 50 mm diameter Hopkinson setup (In.: incident; Tr.: transmitted; SG: strain gauge).

The impact test was carried out using an SHPB device manufactured by Luoyang Levy Technology, Co. (Shanghai, China) As shown in Figure 3, the SHPB device includes a launcher and a test device manufactured by Luoyang Levy Technology, Co. The launcher includes a nitrogen tube, launcher, operating table, barrel, incidence bar, transmission bar, and momentum-absorbing bar. The test device contains a high-strain test system and a laser velocimetry system. The projectile, incidence rod and transmission rod are made of 48CrMoA with *E* = 210 GPa and *ρ* = 7850 kg/m^3^. The relationship between wave velocity and *E* and *ρ* is shown in Equation (11). After calculation, the wave velocity was obtained as 5172.2 m/s.

(11) $$C^{2}=E/\rho$$

**Figure 4:**
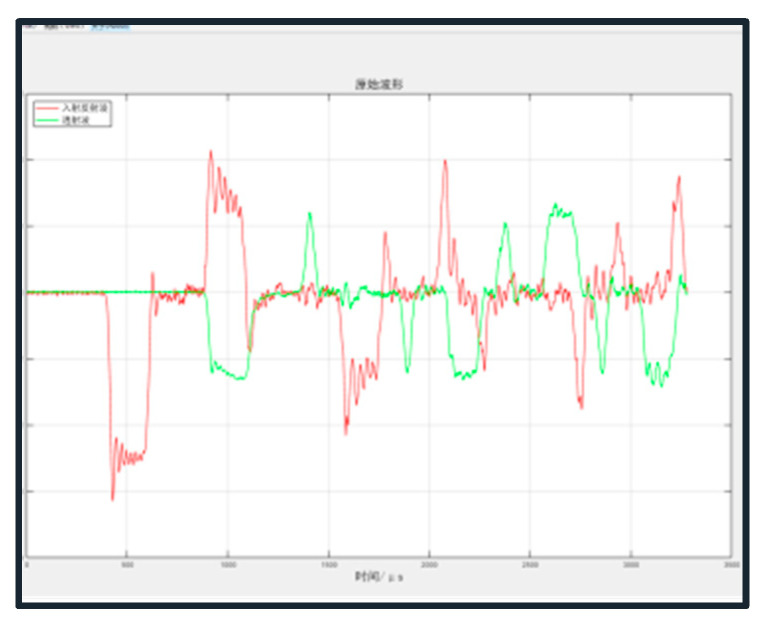
SHPB test data platform.

Before the test, the incident, transmission, and absorption rods were kept at the same horizontal position using Vernier callipers. The sample was coupled to the incident rod interface with petroleum jelly, which served to reduce the effect of test friction on the results. After adjusting the position and setting the impact pressure, the incident rod was affected by the pressure released from the gas gun, producing a one-dimensional stress wave acting on the sample. The collected data were then imported into the test data processing platform to complete the subsequent processing (Figure 4).

### 3.3. Experimental Programme

**Figure 5:**
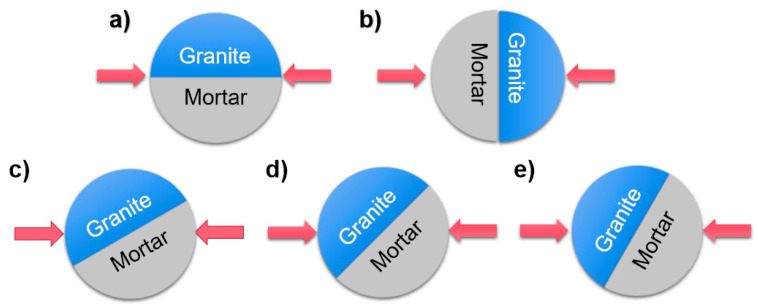
Schematics of the mortar–rock impact loading inclination angles: (**a**) 0°; (**b**) 90°; (**c**) 30°; (**d**) 45°; and (**e**) 60°.

**Figure 6:**
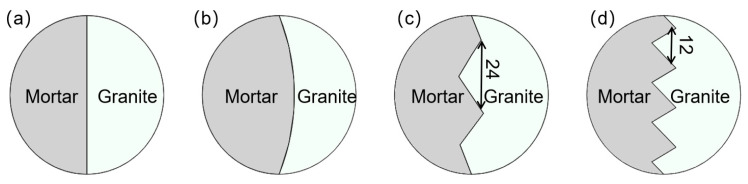
Schematics of the interface shape of mortar–rock specimen: (**a**) straight; (**b**) rounded; (**c**) serrated; and (**d**) multiserrated.

To investigate the effects of the interface on the dynamic fracture mechanical properties of mortar–rock disc materials under impact, the specimens were divided into two groups of working conditions with different shapes and different interface inclination angles. One group had inclination angles of 0°, 90°, 30°, 45°, and 60° (Figure 5). In addition, considering the influence of the interface shape on the dynamic mechanical properties of mortar–rock, four specimens with different interface shapes were produced for dynamic loading testing. By varying the width of the interface serration, four different interface shapes were created: straight (rough), rounded, serrated, and multiserrated (Figure 6). Considering the heterogeneity of the test data, each specimen setup was tested three times to ensure accurate data. The table of test conditions for the mortar–rock binary specimens used for the SHPB tests is presented in Table 5.

### 3.4. Data Reduction

#### 3.4.1. Dynamic Load in the SHPB Test

The dynamic mechanical parameters of the specimen can be calculated indirectly from the measured voltages of the strain gauges in the test: the system provides the specimen with high-strain-rate impact dynamics, with high-speed strain gauges capturing the dynamic incident and reflected waves and transmission rod strain gauges capturing the dynamic transmitted shock waves. The following assumptions must be met to carry out the SHPB test:

(1) The specimen is loaded along the horizontal axial direction, and as an elastic rod, the length of the stress wave far exceeds the diameter of the rod.
(2) A uniformly distributed strain change occurs in the rod along the loading direction.

According to one-dimensional elastic wave theory, the pressure *P* and velocity *V* can be deduced from the signals measured by the strain gauges on both sides, as shown in Equations (12) and (13), respectively.

(12) $$\begin{array}{l} P_{1}\left(t\right)=E\left[\varepsilon_{i}\left(t\right)+\varepsilon_{r}\left(t\right)\right]A\\ V_{1}\left(t\right)=C_{0}\left[\varepsilon_{i}\left(t\right)-\varepsilon_{r}\left(t\right)\right] \end{array}$$

(13) $$\begin{array}{l} P_{2}\left(t\right)=E\varepsilon_{t}\left(t\right)A\\ V_{2}\left(t\right)=C_{0}\varepsilon_{t}\left(t\right) \end{array}$$

where *A* and *E* are the cross-sectional area and elastic modulus of the compressional rod, respectively; $C_{0}$ is the elastic wave velocity of the rod; and *ε_i_*, *ε_r_*, and *ε_t_*_(*t*)_ are the measured incident, reflected, and transmitted wave signals, respectively.

(14) $$\varepsilon_{t}\left(t\right)+\varepsilon_{t}\left(t\right)=\varepsilon_{r}\left(t\right)$$

The incident, reflected, and transmitted wave data are measured by the strain gauges on the incident and transmitted rods, and the stress *σ*, strain *ε*, and strain rate $\overset{\cdot }{\varepsilon }$ of the specimen are deduced. The strain calculation is simplified to the two-wave method, as shown in Equations (15)–(17), for processing and calculation according to expression (3).

(15) $$\sigma =\frac{A_{0}E_{0}}{A_{1}}\varepsilon_{t}\left(t\right)$$

(16) $$\varepsilon =-\frac{2C_{0}}{l_{0}}\int_{0}^{t}\varepsilon_{r}\left(t\right)dt$$

(17) $$\overset{\cdot }{\varepsilon }=-\frac{2C_{0}}{l_{0}}\varepsilon_{t}\left(t\right)$$

where *A*_1_ is the cross-sectional area of the specimen for the BFGRC, and *l*_0_ is the length of the specimen.

**Figure 7:**
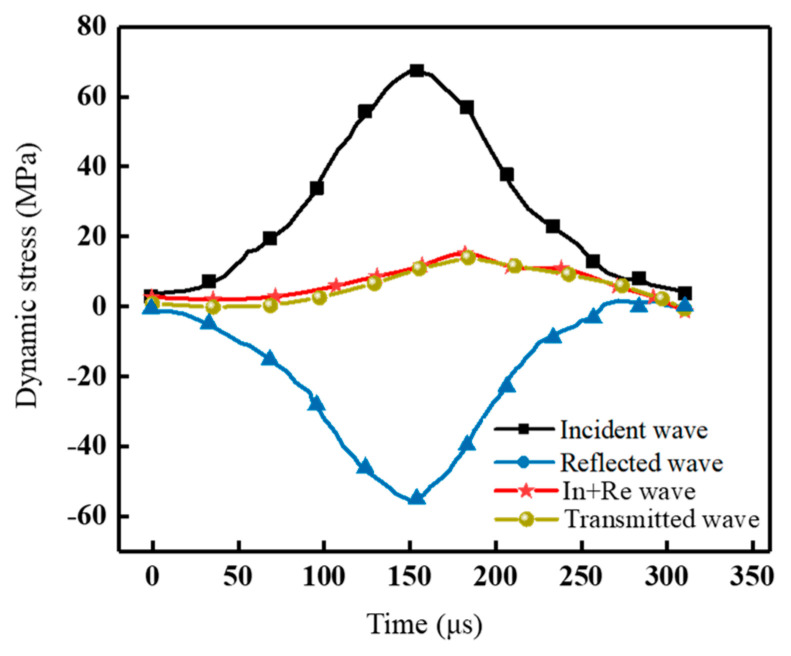
Dynamic stress balance in mortar–rock specimens during the dynamic splitting tests.

From the above equation, it can be seen that if the parameters such as transmissive wave strain, elastic modulus, and cross-sectional area are known, the axial stress of the specimen can be obtained. Based on the strain value and strain rate, the instantaneous axial strain rate of the mortar–rock binary specimen can be deduced. To ensure reliable dynamic mechanical test results, the stress balance needs to reflect that shown in Figure 7. At the sudden event, the dynamic stresses are marked In + Re. The stresses in the figure are the sum of the impact stresses, reflected stresses, and transmitted stresses (the dynamic stresses transferred at the mortar–rock interface). This indicates that the test has achieved dynamic stress equilibrium.

#### 3.4.2. Waveform Shaping

**Figure 8:**
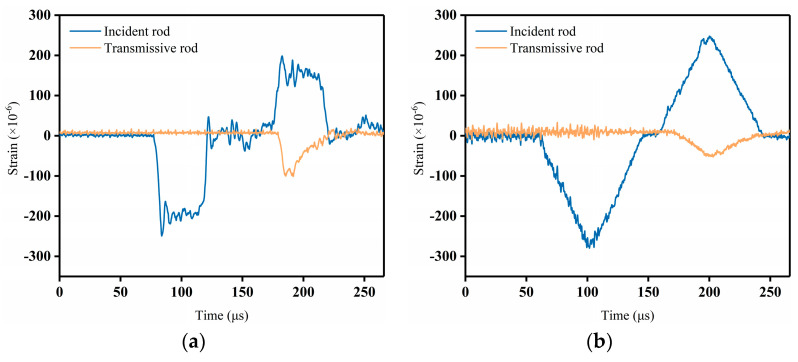
Stress waveforms before and after adjustment: (**a**) before adjustment and (**b**) after adjustment.

In dynamic splitting tests at interfaces, the waveform dispersion effect causes significant oscillations in the actual measured waves. The incident waveform tends to exhibit high-frequency oscillations at short strain rates, and the waveform is trapezoidal, making it difficult to maintain stress equilibrium. As the diameter of the elastic rod and the distance the pulse travels increase, the geometric dispersion effect becomes more pronounced and will affect the assumption of a one-dimensional stress wave. Ensuring a constant strain rate loading is a major challenge to be addressed in dynamic splitting tests at interfaces. To reach stress equilibrium quickly and achieve the desired waveform duration, a brass disc of type H62 brass (with an average copper content of 62% of ordinary brass) was placed between the bullet and the incident rod as a shaper. The brass sheet had a diameter of 20 mm and a thickness of 2 mm. Brass is known for its excellent acoustic properties, such as high sound velocity and low acoustic impedance. When the shock wave generated by the impact reaches the brass sheet, the acoustic properties of the brass help to attenuate and disperse the energy of the shock wave. This diffusion effect reduces the sharpness and intensity of the initial waveform and spreads it over a larger area. The shaper transforms the square wave into a smooth triangular wave by plastic deformation when the impacting rod hits the incident rod at high speed (Figure 8).

## 4. Results and Discussion

### 4.1. Analysis of the Impact Damage Mechanism

**Figure 9:**
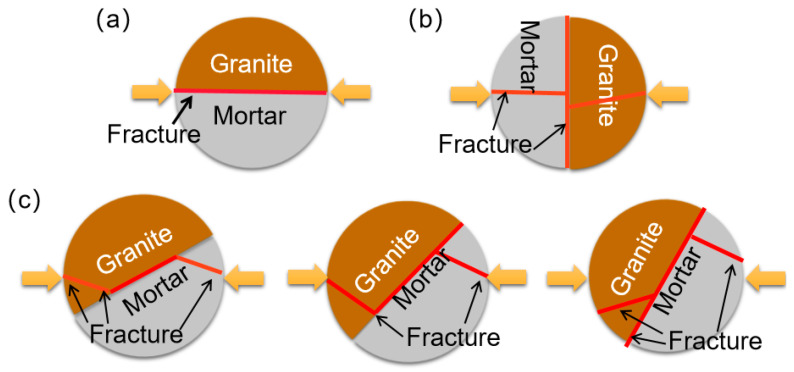
Fracture pattern of the mortar–rock interface for different dip angles: (**a**) interface fracture; (**b**) tensile fracture; and (**c**) composite fracture.

Figure 8 demonstrates the dynamic fracture mechanism of the mortar–rock interface at different interface inclination angles. As shown in Figure 9a, when the interface inclination angle is 0°, the specimens show damage in the form of interface fracture, indicating that the interface is a weak point vulnerable to damage by impact loading. As shown in Figure 9c, when the interface inclination angle is 30°, 45°, and 60°, an interface crack appears as shear damage occurs along the interface; this crack extends into a wing fracture or towards the loading end. Thus, a composite fracture appears. Figure 9b shows that at an interface inclination angle of 90°, the crack pattern of the mortar–rock evolves, extending outwards from the central interface crack to a tensile vertical crack. As the inclination angle increases, the likelihood of tensile cracking and thus damage to the specimen increases. This is due to the decrease in the tensile strength of the mortar–rock binary structure.

### 4.2. Stress–Strain Analysis

**Figure 10:**
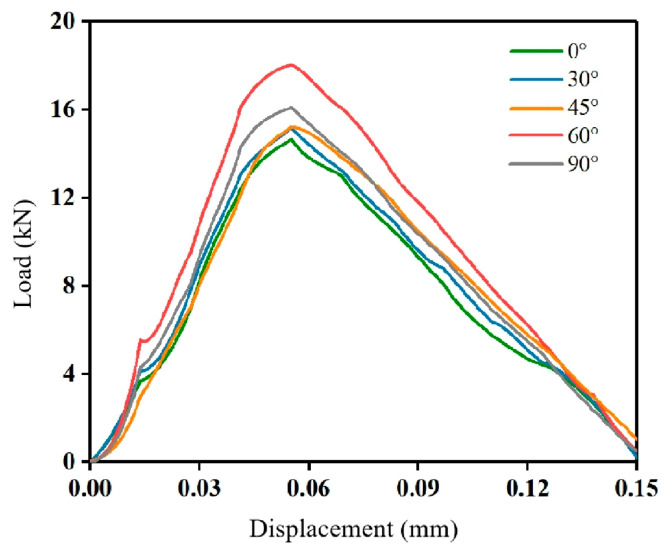
Load–displacement curves for specimens with different inclination angles.

**Figure 11:**
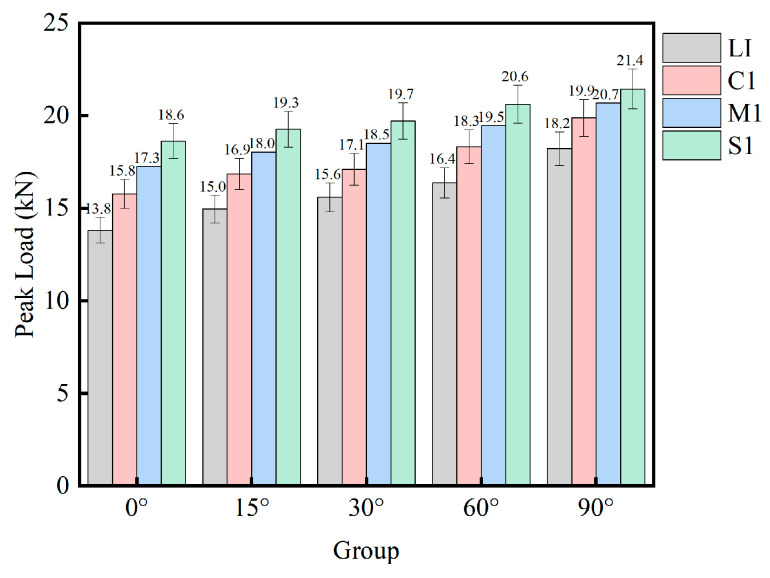
Peak load of specimens with different inclination angles.

The SHPB experimental signals collected by means of strain gauges are imported into the computer by a high-strain tester for processing and analysis. The load—displacement relationship curve is calculated based on the strain recorded by the strain gauge. The dynamic load—displacement curves of the mortar–rock disc specimens under the impact of different inclination angles are shown in Figure 10. The peak load variation pattern of the mortar–rock disc specimens is shown in Figure 11. The results of the analysis show that the dynamic load–displacement curves of the specimens at different interface inclination angles follow almost the same trend. The interface damage under an impact splitting load follows the Mohr–Coulomb (M-C) criterion, and the characteristics of shear damage are observed. When the interface inclination angle is 0°, the specimen fractures along the interface. At this point, the bearing capacity of the mortar–rock specimen is at a low level, and the peak load corresponds to the largest displacement. The smaller the bearing capacity of the mortar–rock disc specimen, the larger the deformation of the specimen. As the inclination of the interface continues to increase, the tensile stress at the interface increases, and the time when the peak load of the specimen arises is continually delayed: the load-bearing performance of the specimen increases. When the interface inclination angle is 90°, the load-bearing capacity reaches its peak. This proves that loading along the interface is the most unfavourable loading method for mortar–rock structures.

**Figure 12:**
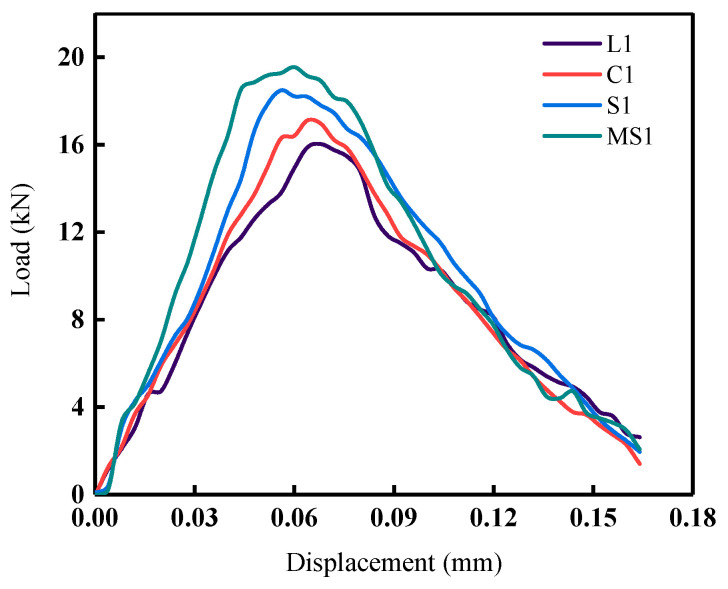
Load–displacement curves for specimens with different interface shapes.

**Figure 13:**
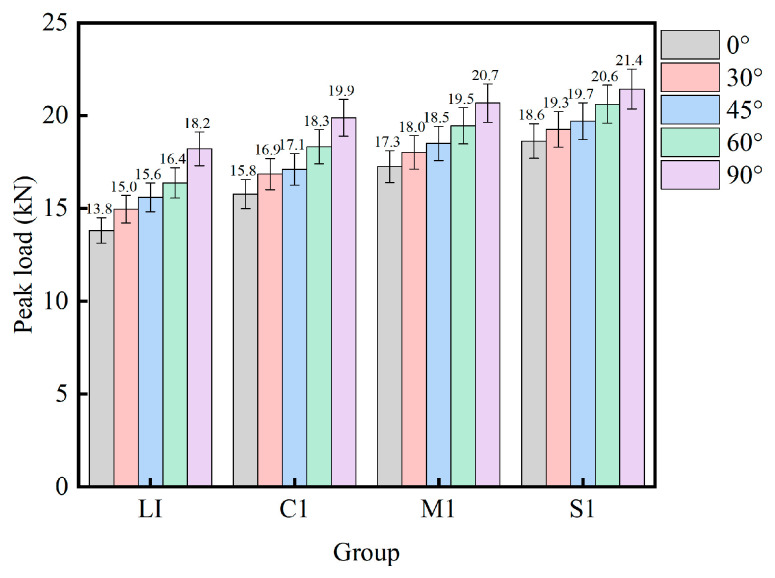
Peak load of specimens with different interface shapes.

The load–displacement response curves of the four interface shapes of mortar–rock binary disc specimens, L1, C1, S1, and MS1, under impact loading are shown in Figure 12. The peak load variation patterns for the four interface shapes are shown in Figure 13. The peak loads for the straight, rounded, serrated, and multiserrated interfaces are 16.03 kN, 17.10 kN, 18.50 kN, and 19.17 kN, respectively. The analysis results illustrate that the smaller the load-bearing capacity of the mortar–rock disc splitting specimen, the larger the deformation of the specimen. The different interface shapes influence the peak loads to which the interface is subjected. The roughness of the interface shape has a greater influence on the dynamic strength of the mortar–rock. The rougher the interface shape, the higher the interfacial joint strength and the higher the peak load that allows the material to reach damage. Of the interface shapes studied, the linear interface is the most susceptible to damage. From Figure 13, it can be seen that as the complexity of the interface shape increases, the peak load difference between the binary specimens at different impact angles (0°, 90°) gradually decreases. This indicates to some extent that the influence of the interface shape on the fracture resistance of the specimen interface is greater than that of the interface angle. The results show that a change in interface shape can significantly improve the dynamic load-bearing performance of the mortar–rock binary material. 

### 4.3. Energy Absorption Analysis

In the interface dynamic splitting test, the incident energy is converted into the deformation energy of the specimen, and the energy conservation criterion is met. When the incident energy is constant, specimens with different interfacial crack inclinations have different load-bearing capacities. Combined fracture (shear and tensile) occurs when the angle of impact is 30–60°. The amount of energy absorbed is defined as the energy absorption density (w), which is a measure of the ability of concrete to resist breaking under impact loading. The energy absorption density can be calculated according to Equation (18).

(18) $$w=J_{0}\left(c\right)ds$$

**Figure 14:**
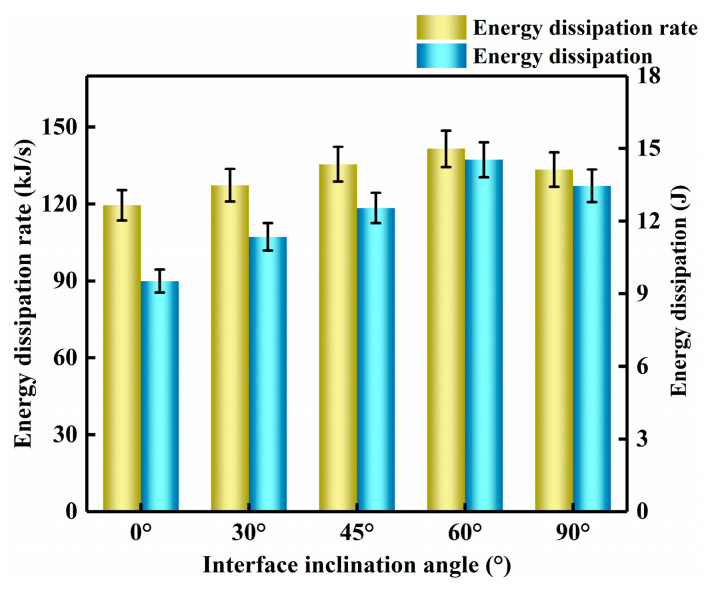
Energy dissipation and energy dissipation rate at the mortar–rock interface for different inclination angles.

For a linear specimen interface shape, Figure 14 indicates the energy density and energy dissipation rate of mortar–rock specimens with different interface inclination angles. When the inclination angle is 0°, the energy density and energy dissipation rate are the lowest. Compared with 0°, when the specimen interface inclination angles were 30°, 45°, 60°, and 90°, the energy dissipation increased by 15.9%, 31.7%, 52.6%, and 41.3%, and the energy dissipation rate increased by 6.6%, 13.4%, 18.4%, and 11.7% (Figure 14). These results show that the dissipated energy and dissipation rate gradually increase to a peak in the range of 0° to 60° as the interface inclination increases. The dissipation energy of the specimens with an inclination of 90° decreases slightly compared with that at an interface inclination of 60°. When loaded along the mortar–rock interface, the binary specimens fracture along the interface. At this point, the specimen has the lowest tensile strength, resulting in the lowest dissipation energy and dissipation energy rate. Interfacial fracture is caused by shear fracture of the specimen. Shear fracture requires less energy to be expended than tensile fracture [34,44]. Due to the weak strength of the interface, fractures can easily form along the interface. In this situation, the peak load is the lowest, and the dissipated energy is minimum as well. A study of the trend in the movement of the I/II composite fracture of the binary specimen revealed a slowing down of the crack extension in the crack deflection region of the specimen. This phenomenon is a sign of fracture mode transition. The results of this study are in general agreement with the findings of Zhou [34] and Guo et al. [37].

**Figure 15:**
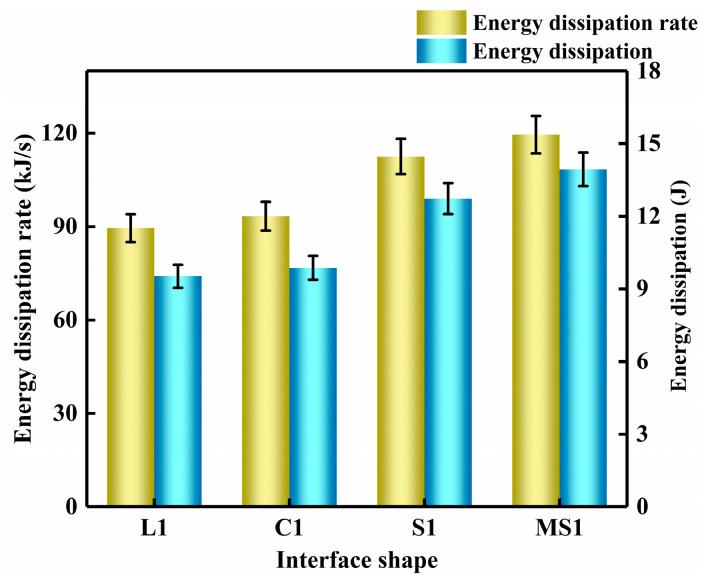
Energy dissipation and energy dissipation rate at the mortar–rock interface with different shapes.

Figure 15 shows the energy density and energy dissipation rate of the mortar–rock specimens for different interface shapes. The energy dissipation increases by 3.6%, 33.6%, and 46.3% when the interface shape is rounded, serrated, and multiserrated compared with the linear shape, and the energy dissipation rates increase by 4.3%, 25.7%, and 33.5%, respectively. Analysis of the data shows that when the interface is linear, the interface joint strength is relatively low and cannot guarantee sufficient tensile strength; therefore, the relative energy dissipation and energy dissipation rate of the specimen are small in that case. When the interface is rounded, the tensile strength is nearly similar to that of the linear type. In contrast, the sawtooth shape has a significantly higher tensile strength due to its better occlusion. Overall, there is a consistent pattern of change in the energy dissipation and energy rate of the interface. This is due to the higher dynamic loading speed.

### 4.4. Splitting Tensile Strength

**Figure 16:**
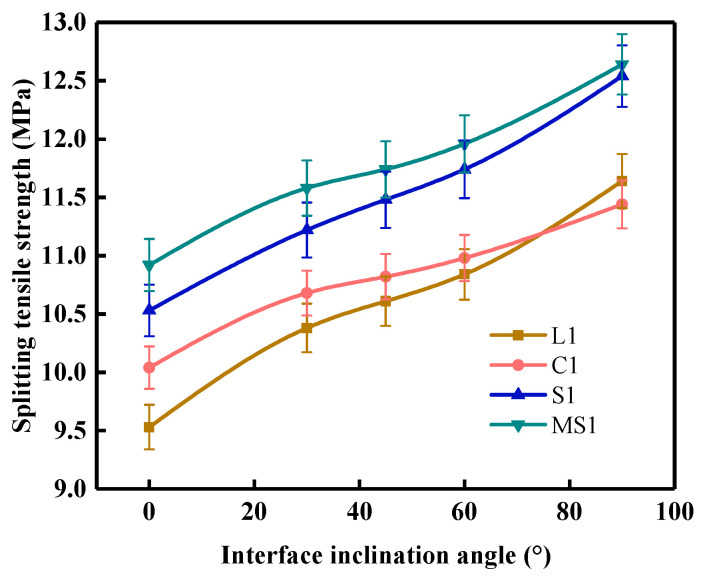
Dynamic splitting strength of mortar–rock binary specimens.

The dynamic splitting strength of the mortar–rock specimens is shown in Figure 16. It can be seen from the figure that the dynamic splitting strength of the specimen is the smallest when the interface inclination angle is 0°. As the interface inclination angle increases, the dynamic splitting strength of the mortar–rock material continues to increase. When the interface inclination angle is in the range of 0–30°, the dynamic tensile strength of the specimen increases faster. At this point, the interface undergoes not only damage in the direction of the interface but also tensile-type damage off to one side, showing a trend of composite fracture. In the range of 30–60°, the dynamic tensile strength increases more slowly. In the range of 60–90°, the specimen interface damage gradually decreases and changes from composite fracture to tensile fracture when the tensile strength of the specimen reaches its peak. The results show that a specimen undergoes a process of damage first increasing and then decreasing. To prevent the mortar–rock structure from cracking at the interface under impact loading, more consideration should be given to the occurrence of tensile fractures in the material and avoiding impact to interfaces at angles of 0–60° as much as possible to improve the impact resistance of the structure.

### 4.5. Interface Dynamic Model

**Figure 17:**
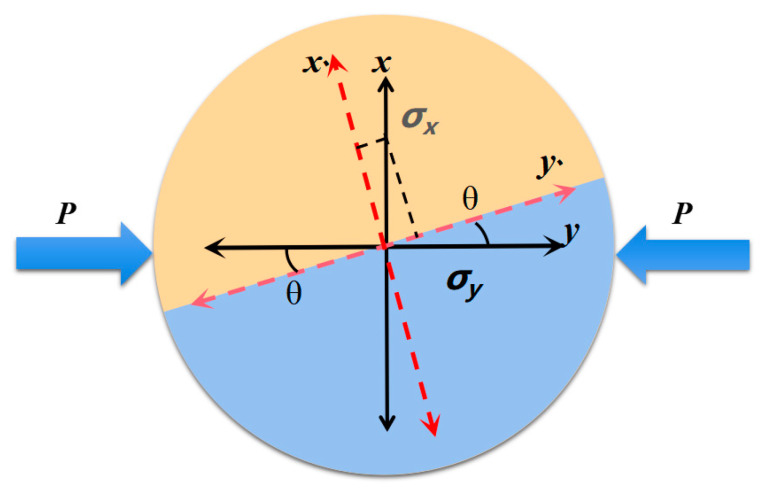
Physical model of a mortar–rock binary specimen.

The dynamic mechanical model of mortar–granite binary specimens is shown in Figure 17. For the analysis of the mortar–rock binary physical model, the tensile stresses in the vertical loading direction and the compressive stresses along the loading direction are *σ_x_* and *σ_y_*, respectively, as shown in Equations (19) and (20)

(19) $$\sigma_{x^{\prime }}=\sigma_{x}\cos\theta -\sigma_{y}\sin\theta$$

(20) $$\sigma_{y^{\prime }}=\sigma_{y}\cos\theta -\sigma_{x}\sin\theta$$

The ratio of tensile stress to shear stress *f_s_* is shown in Equation (21), and the calculated results of mortar–rock under different interface inclination angles are shown in Table 6.

(21) $$f_{s}=\frac{\sigma_{y^{\prime }}}{\sigma_{x^{\prime }}}=\frac{\sigma_{y}\cos\theta -\sigma_{x}\sin\theta }{\sigma_{x}\cos\theta -\sigma_{y}\sin\theta }=\frac{3\cos\theta +\sin\theta }{\cos\theta -3\sin\theta }$$

Dynamic damage at the mortar–rock interface under impact loading follows the M-C criterion [51]. Table 6 shows the variation in fs for mortar–rock binary specimens with different interface inclination angles. When the inclination angle is 0°, 30°, 45°, 60° and 90°, *f_s_* is 30°, 45°, 60° and 90°, respectively. As the interface inclination angle continues to increase, the stress state at the interface gradually changes from shear stress to tensile stress, accompanied by a change in the damage mode of the specimen from shear damage to tensile damage. The likelihood of tensile cracking damage increases. During mixed mode I/II crack extension, a deceleration in the rate of crack extension occurs in the deflection zone of the mortar–rock crack path, which is a sign of a fracture mode shift. Under tensile stress, the mortar–rock binary specimen is damaged by the tensile fracture in the centre of the disc, progressing towards the loading direction until the entire specimen is split into two half-discs.

## 5. Conclusions

In this paper, a theoretical model of mortar–rock binary impact was established based on the theory of interfacial fracture mechanics. Impact mechanics tests at the mortar–rock interface were carried out with an SHPB device, focusing on the properties of the mortar–rock binary with different interface inclinations and interface roughnesses under dynamic compression, and the following conclusions were obtained:

1. Three fracture modes were observed in the mortar–rock binary specimens: type I fracture, type II fracture, and mixed type I/II fracture. At high strain rates, stress concentrations usually accelerate damage near the loaded end rather than at the centre of the specimen. At an interface inclination of 0°, the specimen exhibits shear damage with an interface fracture. When the interface dip angle is 0–90°, the interface cracks via shear damage along the interface, at which point the mortar–rock binary specimen fractures in a composite manner. When the interface inclination angle is 90°, the crack pattern of the mortar–rock evolves, extending outwards from the central interface crack and evolving from composite fracture to tensile fracture.
2. As the interface inclination angle increases, the dissipation energy and dissipation energy rate gradually increase to a peak in the inclination angle range of 0° to 60°. In comparison, the dissipation energy of specimens with an interface inclination of 90° decreases slightly. The reason for this pattern is that when the loading direction is along the mortar–rock interface, the binary specimens fracture along the interface. At this point, the specimen has the lowest tensile strength, resulting in the lowest dissipation energy and dissipation energy rate. Interface fracture, on the other hand, is caused by shear fracture of the specimen along the interface, and shear fracture requires more energy to be dissipated than tensile fracture.
3. The interface inclination angle has an effect on the splitting tensile strength of the rock–mortar disc material. As the interface inclination angle increases, the dynamic splitting strength of the mortar–rock material increases. When the interface inclination is in the range of 30–60°, the dynamic tensile strength increases more slowly. In the range of 60–90°, the interface damage gradually decreases, and the dynamic tensile strength of the specimen reaches its peak. During mixed mode I/II crack expansion, a deceleration of crack expansion occurs in the deflection zone of the crack path, which is a sign of a fracture mode shift.
4. Different interface shapes influence the dynamic mechanical properties of the mortar–rock material. The roughness of the interface shape has a large influence on the dynamic strength of the mortar–rock. The rougher the interface shape, the higher the interface cleavage strength and the higher the peak load that allows the material to reach damage. Of the four shapes, the linear interface is the most susceptible to damage. Adequate tensile strength cannot be guaranteed when the interface is linear. The relative dissipation energy and dissipation energy rate of the specimen are small. When the interface is rounded, the tensile strength is similar to that of a straight specimen. The serrated shape corresponds to a significant increase in tensile strength due to better occlusion. The complexity of the interface shape significantly improves the dynamic load-bearing performance of the mortar–rock binary material.
5. The damage of the mortar–rock interface under impact loading follows the M-C criterion. With increasing inclination of the interface, the stress state of the interface gradually changes from shear stress to tensile stress, accompanied by a change in the damage mode of the specimen from shear damage to tensile damage. The likelihood of tensile cracking damage increases. Eventually, the mortar–rock binary specimen is damaged by the tensile stress.

## Figures and Tables

**Table 1:** Physical properties of the aggregates.

Type of Aggregate	Compressive Strength/MPa	Elastic Modulus /MPa	Apparent Density /kg·m^−3^	Saturated Surface Water Absorption/%	ω/%
Granite	150	70	2850	0.46	12.8

**Table 2:** Components of the mortar.

Grade	Water kg/m^3^	Cement kg/m^3^	Sand kg/m^3^	Silica Fume /%	Additives /%
M10	280	250	1125	5	2

**Table 3:** Basic physical and mechanical parameters of cement.

Property	Density /g·cm^−3^	Compressive Strength/MPa		Flexural Strength /MPa		Setting Time /min		Standard Consistency
		3d	7d	3d	7d	3d	7d	
Value	3.23	29.2	45.1	5.2	8.4	135	205	30

**Table 4:** Components of the granite.

Group	Quartz	Albite	Microcline	Biotite
Proportion	46.78	31.39	20.61	1.22

**Table 5:** SHPB test conditions.

Group	Specimen Size (mm)	Interface Shape	Impact Loading Angle (°)
L1	Φ48 mm × 25 mm	Straight	0°
L2			30°
L3			45°
L4			60°
L5			90°
CI		Rounded	0°
C2			30°
C3			45°
C4			60°
C5			90°
S1		Serrated	0°
S2			30°
S3			45°
S4			60°
S5			90°
MS1		Multiserrated	0°
MS2			30°
MS3			45°
MS4			60°
MS5			90°

**Table 6:** Mortar–rock *f_s_* at different loading angles.

Angle	0°	30°	45°	60°	90°
*f_s_*	3.21	−4.86	−3.57	−2.46	−1.37

## Data Availability

The data presented in this study are available on request from the corresponding author.

## References

[1] Dong J., Li Z.Q., Yan X., Liu Y., Zhao S.R., Qian R., Zheng Y.H., Jiang T. (2022). Effects of coarse aggregates on the mechanical properties, durability and microscopic behaviour of mortar rubble. Constr. Build. Mater..

[2] Zhu W.C., Bai Y., Li X.B., Niu L.L. (2012). Numerical simulation on rock failure under combined static and dynamic loading during SHPB tests. Int. J. Impact Eng..

[3] Bai Y.l., Yan Z.W., Jia J.F., Ozbakkaloglu T., Liu Y. (2021). Dynamic compressive behavior of concrete confined with unidirectional natural flax FRP based on SHPB tests. Compos. Struct..

[4] Guo Y.B., Gao G.F., Jing L., Shim V.P.W. (2017). Response of high-strength concrete to dynamic compressive loading. Int. J. Impact Eng..

[5] Yu Q., Zhuang W., Shi C. (2021). Research progress on the dynamic compressive properties of ultra-high performance concrete under high strain rates. Cem. Concr. Compos..

[6] Zhang H., Wang L., Zheng K., Bakura T.J., Totakhil P.G. (2018). Research on compressive impact dynamic behavior and constitutive model of polypropylene fiber reinforced concrete. Constr. Build. Mater..

[7] Kim K.M., Lee S., Cho J.Y. (2022). Influence of friction on the dynamic increase factor of concrete compressive strength in a split Hopkinson pressure bar test. Cem. Concr. Compos..

[8] Sun B., Chen R., Ping Y., Zhu Z., Wu N., Shi Z. (2022). Research on dynamic strength and inertia effect of concrete materials based on large-diameter split hopkinson pressure bar test. Materials.

[9] Han Z., Xie S., Li D., Zhu Q., Yan Z. (2022). Dynamic mechanical behavior of rocks containing double elliptical inclusions at various inclination angles. Theor. Appl. Fract. Mech..

[10] Wu L., Huang D. (2022). Peridynamic modeling and simulations on concrete dynamic failure and penetration subjected to impact loadings. Eng. Fract. Mech..

[11] Zhou X.Q., Hao H. (2008). Mesoscale modelling of concrete tensile failure mechanism at high strain rates. Comput. Struct..

[12] Bi J., Liu P., Gan F. (2020). Effects of the cooling treatment on the dynamic behavior of ordinary concrete exposed to high temperatures. Constr. Build. Mater..

[13] Jin L., Yu W., Du X., Yang W. (2019). Mesoscopic numerical simulation of dynamic size effect on the splitting-tensile strength of concrete. Eng. Fract. Mech..

[14] Jin L., Yu W., Du X., Zhang S., Li D. (2019). Meso-scale modelling of the size effect on dynamic compressive failure of concrete under different strain rates. Int. J. Impact Eng..

[15] Li Z.Q., Chen H., Dong J., Yan X., Zhao S.R., Zheng Y.H., Liu Y. (2022). Study of the failure mechanism of mortar rubble using digital image correlation, acoustic emission and scanning electron microscopy. Buildings.

[16] Qiu H., Zhu Z., Wang M., Wang F., Ma Y., Lang L., Ying P. (2020). Study on crack dynamic propagation behavior and fracture toughness in rock-mortar interface of concrete. Eng. Fract. Mech..

[17] Mouzannar H., Bost M., Leroux M., Virely D. (2017). Experimental study of the shear strength of bonded concrete–Rock interfaces: Surface morphology and scale effect. Rock Mech. Rock Eng..

[18] Ping Q., Shen K., Gao Q., Wang C., Wang S., Wu Y., Hu J. (2022). Experimental study on dynamic performance of rock-concrete composite with different thickness ratios. Shock Vib..

[19] Gao H., Zhai Y. (2022). Numerical investigation of the concrete–rock combined body influence of inclined interface on dynamic characteristics and failure behaviors. Arab. J. Geosci..

[20] Jia J.Y., Gu X.L. (2021). Effects of coarse aggregate surface morphology on aggregate-mortar interface strength and mechanical properties of concrete. Constr. Build. Mater..

[21] Chang X., Guo T., Lu J., Wang H. (2019). Experimental study on rock-concrete joints under cyclically diametrical compression. Geomech. Eng..

[22] Kaplan M.F. (1961). Crack propagation and the fracture of concrete. J. Proc..

[23] Qiu H., Zhu Z., Wang M., Wang F., Luo C., Wan D. (2020). Study of the failure properties and tensile strength of rock-mortar interface transition zone using bi-material Brazilian discs. Constr. Build. Mater..

[24] Huang K., Guo L., Yu H., Jia P., Kitamura T. (2016). A domain-independent interaction integral method for evaluating the dynamic stress intensity factors of an interface crack in nonhomogeneous materials. Int. J. Solids Struct..

[25] Chang J., Xu J.Q. (2007). The singular stress field and stress intensity factors of a crack terminating at a bimaterial interface. Int. J. Mech. Sci..

[26] Tong J., Wong K.Y., Lupton C. (2007). Determination of interfacial fracture toughness of bone–cement interface using sandwich Brazilian disks. Eng. Fract. Mech..

[27] Dong W., Yang D., Zhang B., Wu Z. (2018). Rock-concrete interfacial crack propagation under mixed mode I–II fracture. J. Eng. Mech..

[28] Dong W., Wu Z., Zhou X. (2016). Fracture mechanisms of rock-concrete interface: Experimental and numerical. J. Eng. Mech..

[29] Zhou X., Xie Y., Long G., Zeng X., Li J., Yao L., Jiang W., Pan Z. (2021). DEM analysis of the effect of interface transition zone on dynamic splitting tensile behavior of high-strength concrete based on multi-phase model. Cem. Concr. Res..

[30] Gui Y.L., Bui H.H., Kodikara J., Zhang Q.B., Zhao J., Rabczuk T. (2016). Modelling the dynamic failure of brittle rocks using a hybrid continuum-discrete element method with a mixed-mode cohesive fracture model. Int. J. Impact Eng..

[31] Wang L., Wang L., Yang Y., Zhu X., Zhang D., Gao X. (2022). Discrete element modeling of rock-concrete bi-material discs under dynamic tensile loading. Constr. Build. Mater..

[32] Han Z., Li D., Li X. (2022). Dynamic mechanical properties and wave propagation of composite rock-mortar specimens based on SHPB tests. Int. J. Min. Sci. Technol..

[33] Zhou Z., Lu J., Cai X. (2020). Static and dynamic tensile behavior of rock-concrete bi-material disc with different interface inclinations. Constr. Build. Mater..

[34] Guo T., Liu K., Ma S., Yang J., Li X., Zhou K., Qiu T. (2022). Dynamic fracture behavior and fracture toughness analysis of rock-concrete bi-material with interface crack at different impact angles. Constr. Build. Mater..

[35] Zhong Z.B., Deng R.G., Zhang J., Hu X.Z. (2020). Fracture properties of jointed rock infilled with mortar under uniaxial compression. Eng. Fract. Mech..

[36] Wang W.C., Sun S.R., Le H.L., Shu Y., Zhu F., Fan H.T., Liu Y. (2019). Experimental and numerical study on failure modes and shear strength parameters of rock-like specimens containing two infilled flaws. Int. J. Civ. Eng..

[37] Guo Y., Chen X., Wang Z., Ning Y., Bai L. (2023). Identification of mixed mode damage types on rock-concrete interface under cyclic loading. Int. J. Fatigue.

[38] Chen X., Hu L., Feng Z., Guo Y., Ning Y., Xue X. (2022). Dynamic splitting tensile behavior of rock–concrete bimaterial disc with multiple material types under different interface inclination angle. Fatigue Fract. Eng. Mater. Struct..

[39] Dong W., Song S., Zhang B., Yang D. (2019). SIF-based fracture criterion of rock-concrete interface and its application to the prediction of cracking paths in gravity dam. Eng. Fract. Mech..

[40] Luo L., Li X., Tao M., Dong L. (2017). Mechanical behavior of rock-shotcrete interface under static and dynamic tensile loads. Tunn. Undergr. Space Technol..

[41] Mpalaskas A.C., Matikas T.E., Van Hemelrijck D., Iliopoulos S., Papakitsos G.S., Aggelis D.G. (2015). Acoustic signatures of different damage modes in plain and repaired granite specimens[C]//Smart Materials and Nondestructive Evaluation for Energy Systems 2015. SPIE.

[42] Liu K., Guo T., Yang J., Ma S. (2023). Static and dynamic fracture behavior of rock-concrete bi-material disc with different interface crack inclinations. Theor. Appl. Fract. Mech..

[43] Zhu J., Bao W., Peng Q., Deng X. (2020). Influence of substrate properties and interfacial roughness on static and dynamic tensile behaviour of rock-shotcrete interface from macro and micro views. Int. J. Rock Mech. Min. Sci..

[44] Selçuk L., Aşma D. (2019). Experimental investigation of the rock–concrete bi materials influence of inclined interface on strength and failure behavior. Int. J. Rock Mech. Min. Sci..

[45] Qiu J.D., Li D.Y., Li X.B., Zhu Q.Q. (2020). Numerical investigation on the stress evolution and failure behavior for deep roadway under blasting disturbance. Soil Dyn. Earthq. Eng..

[46] Gutiérrez-Ch J.G., Senent S., Melentijevic S., Jimenez R. (2018). Distinct element method simulations of rock-concrete interfaces under different boundary conditions. Eng. Geol..

[47] Qiu H., Wang F., Zhu Z., Wang M., Yu D., Luo C., Wan D. (2021). Study on dynamic fracture behaviour and fracture toughness in rock-mortar interface under impact load. Compos. Struct..

[48] Dong S., Wang Y., Xia Y. (2004). Stress intensity factors for central cracked circular disk subjected to compression. Eng. Fract. Mech..

[49] Ayatollahi M.R., Aliha M.R.M. (2007). Fracture toughness study for a brittle rock subjected to mixed mode I/II loading. Int. J. Rock Mech. Min. Sci..

[50] Chang S.H., Lee C.I., Jeon S. (2002). Measurement of rock fracture toughness under modes I and II and mixed-mode conditions by using disc-type specimens. Eng. Geol..

[51] Liu P., Zhou X., Qian Q., Berto F., Zhou L. (2020). Dynamic splitting tensile properties of concrete and cement mortar. Fatigue Fract. Eng. Mater. Struct..

